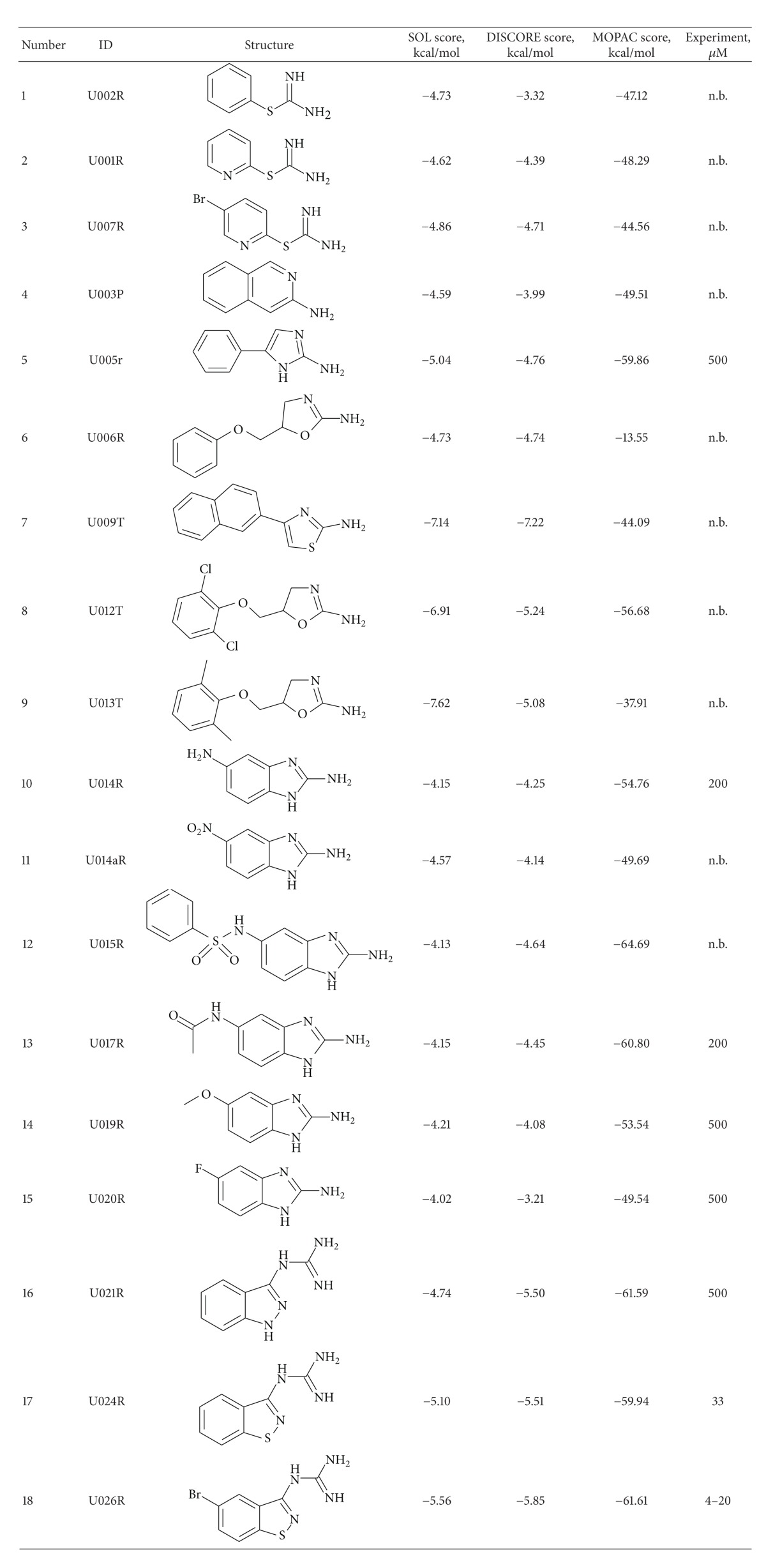# Application of Molecular Modeling to Urokinase Inhibitors Development

**DOI:** 10.1155/2014/625176

**Published:** 2014-05-20

**Authors:** V. B. Sulimov, E. V. Katkova, I. V. Oferkin, A. V. Sulimov, A. N. Romanov, A. I. Roschin, I. B. Beloglazova, O. S. Plekhanova, V. A. Tkachuk, V. A. Sadovnichiy

**Affiliations:** ^1^Research Computer Center, Moscow State University, Leninskie Gory 1, Building 4, Moscow 119992, Russia; ^2^Dimonta Ltd., Nagornaya Street 15, Building 8, Moscow 117186, Russia; ^3^Cardiology Research Center of Russian Ministry of Health 3rd Cherepkovskaia, No. 15a, Moscow 121552, Russia; ^4^Faculty of Medicine, Lomonosov Moscow State University, Lomonosovskiy Prospect 31, Building 5, Moscow 119192, Russia; ^5^Lomonosov Moscow State University, Leninskie Gory 1, Moscow 119991, Russia

## Abstract

Urokinase-type plasminogen activator (uPA) plays an important role in the regulation of diverse physiologic and pathologic processes. Experimental research has shown that elevated uPA expression is associated with cancer progression, metastasis, and shortened survival in patients, whereas suppression of proteolytic activity of uPA leads to evident decrease of metastasis. Therefore, uPA has been considered as a promising molecular target for development of anticancer drugs. The present study sets out to develop the new selective uPA inhibitors using computer-aided structural based drug design methods. Investigation involves the following stages: computer modeling of the protein active site, development and validation of computer molecular modeling methods: docking (SOL program), postprocessing (DISCORE program), direct generalized docking (FLM program), and the application of the quantum chemical calculations (MOPAC package), search of uPA inhibitors among molecules from databases of ready-made compounds to find new uPA inhibitors, and design of new chemical structures and their optimization and experimental examination. On the basis of known uPA inhibitors and modeling results, 18 new compounds have been designed, calculated using programs mentioned above, synthesized, and tested *in vitro*. Eight of them display inhibitory activity and two of them display activity about 10 **μ**M.

## 1. Introduction: Role and Structure of Urokinase-Type Plasminogen Activator (uPA)

The key principle of the rational drug development is to determine the compound, which blocks the functioning protein responsible for the progression of the disease. Thus, the central problem of the new drug development is to find the organic compound (the inhibitor) capable to bind selectively to a given target-protein. At present, search of new inhibitors is a crucial step of drug discovery and it takes about 50% of the total time of new remedies development. For a long time, experimental screening of hundreds of thousands of organic test compounds from chemical libraries was the main search strategy of new drugs design. However, lately molecular modeling computational methods are getting involved in the work [1], and they can significantly reduce the cost and material resources.

The present paper is devoted to the detailed account of the application of molecular modeling to search and design of the novel antitumor drug on the base of uPA proteolytic site inhibitors.

The data accumulated for the last years show that uPA system has a lot of functions in the evolution malignant tumors, including the angiogenesis regulation (including the tumor angiogenesis), cell division, adhesion, and migration of the malignant cells and tumor metastasis [2–4]. Normally injury stimulates the local urokinase (uPA) expression [5]. That makes sense because the vessels are traumatized, and haemorrhage and blood clot organization happen upon damages. For this reason, it was supposed that urokinase is a key element in the vessel and tissues remodeling and regeneration processes. Experimental data also suggest urokinase participation in the regulation of the cell migration and proliferation [2, 4].

Pathogenic processes use the same molecules and mechanisms which operate under normal conditions. Main tumor responsible mechanisms are cell proliferation and migration. Vessel growth is the necessary condition for the tumor growth. The uPA test is considered now as one of promising markers for the breast cancer prognosis and treatment.

Due to urokinase participation in tumor development its components can be considered as an appropriate target for anticancer therapy [6]. Several studies indicate the potential efficiency of the therapeutic approaches targeted inhibition of the uPA system components expression or inhibition of the uPA activity itself or inhibition of its interaction with receptor with the purpose of suppressing of the tumor growth [7–9].

The uPA is a 53 kDa multidomain glycoprotein of 411 residues. The N-terminal (A-chain) contains the kringle domain and the epidermal growth factor (EGF)-like domain; the latter responsible for the binding to uPAR, whereas the C-terminal (B-chain) is composed of two subdomains formed by six beta strands folded in an antiparallel manner and connected by twist or helical regions. The active site is located at the interface between the two subdomains, and consists of the catalytic triad of His57, Asp102, and Ser195 residues [10].

At present, there is a large amount of data on the mechanism of urokinase action on angiogenesis and remodeling, urokinase and its receptor atomic structures, and knowledge of the mechanism of urokinase and the receptor interaction. So, all necessary conditions are presented for considering uPA as a challenging target for the therapeutic action of a new antitumor drug, and computer molecular modeling techniques can be applied for development of new compounds that suppressed certain functions of uPA.

Function and activity regulation of the proteolytic domain of uPA are known better than the functions of other uPA domains. Importance of proteolytic activity of uPA in the tumor growth and metastasis was demonstrated repeatedly [2, 3, 11–13]. Thereby development of uPA proteolytic activity inhibitors is presently one of the most actual and well-defined problems.

The natural urokinase plasminogen activator (uPA) and tissue plasminogen activator (tPA) inhibitors in human organism are PAI-1 and PAI-2 proteins, which form covalent bonds with uPA and tPA after binding and inactivate uPA and tPA irreversibly, which results in the fibrinolysis suppression due to decrease of plasminogen activity. This is the way hemorrhage by fibrinolysis in the organism is prevented.

The first evidence that disclosed the possibility of arresting tumor progression by means of inhibitors of the uPA activity was achieved with anti-uPA antibodies [14, 15]. Subsequently, the efforts in this field have been directed to the development of small-molecule synthetic uPA inhibitors with appropriate potency, selectivity, and pharmacokinetic properties for use as anticancer drugs.

Specificity of binding toward basic substrates is largely determined by the residue Asp189, located at the base of the primary anchor site for substrates, termed specificity pocket S1 [16]. The crystal structure of uPA catalytic domain displays a trypsin-like topology in which the Asp189 is retained, conferring to the S1 site an affinity for positively charged Arg and Lys residues [10]. Therefore, the majority of synthetic uPA inhibitors, conceived so far, share a common structural feature consisting of a mono- or biaromatic moiety substituted with an amidino or guanidino function, acting as arginine mimetic. However, a strong limitation in the choice of feasible compounds is represented by the necessity to inhibit uPA without affecting the activity of other trypsin-like serine proteases, and especially tPA and plasmin, essential for the fibrinolytic processes.

Earliest works were recognized as selective uPA inhibitors, the para-substituted benzamidine derivatives, the 4-chloro- and 4-trifluoromethylphenylguanidines, and amiloride, but none of them was endowed of high potency and all *K*_*i*_ values fell within the micromolar range [17–19]. The research of more powerful compounds led to the synthesis of two 4-substituted benzo[b]thiophene-2-carboxamidines, B428 and B623, with *K*_*i*_ of 0.53 and 0.16 *μ*M, respectively [20]. In the past few years, a number of novel small-molecule uPA inhibitors have been proposed, including the 4-aminoarylguanidine and 4-aminobenzamidine derivatives, 2-pyridinylguanidines, 1-isoquinolinylguanidines and 1-(7-sulfonamidoisoquinolinyl)guanidines, mexiletine derivatives, the 4-chloro-3-alkoxyisocoumarins containing a terminal bromine, the 4-oxazolidinone analogue UK122, 5-thiomethylthiopheneamidine derivatives, the N-(1-adamantyl)-N′-(4-guanidinobenzyl)urea (WX-293T), and WX-UK1, a derivative of 3-amidinophenylalanine [21–31]. However, among these inhibitors, only WX-UK1 (WILEX, Munich, Germany) entered clinical development, showing a *K*_*i*_ against human uPA of 0.6 *μ*M. Since WX-UK1 is not absorbed orally, more recently, Wilex developed an oral prodrug, WX-671, for the systemic delivery of the active WX-UK1. This prodrug is currently under evaluation in two independent studies of phase II clinical trials in combination with classical cytotoxic treatments to estimate its efficiency [6]. Nevertheless, there is no drug based on the uPA inhibitor to the date, integrated in the clinical practice, so the design of such inhibitors is now an actual problem.

The present paper summarizes the experience of the rational design of new synthetic low-molecular uPA inhibitors using computer molecular modeling techniques. This work also describes the technique of the target-protein (urokinase) molecular model building and the used modeling techniques: docking for ligand positioning into the active site of the target-protein by the global protein-ligand potential energy minimum search, postprocessing for refinement of the free binding energy of the protein-ligand system, generalized direct docking for refinement of the free binding energy of the protein-ligand system by including in calculations not only the global minimum, but also the nearby local energy minima and by computing not only the enthalpy component but also the entropy component of the free binding energy accounting the oscillations of the ligand atoms, and the quantum chemistry techniques for the more accurate calculation of the protein-ligand interaction energy. This paper also presents the results of validation of few techniques for native urokinase ligands, describes the virtual screening of databases of ready-made compounds for uPA inhibitors, and depicts the results of the new uPA inhibitors development.

## 2. Methods

### 2.1. Molecular Model of Urokinase Target-Protein

The input data to begin the drug design using molecular modeling methods are the three-dimensional coordinates of the targeted protein. More than 60 structures contained one or another uPA domain were found in the Protein Data Bank (PDB) [32] database as the result of the respective search.

**Figure 1:**
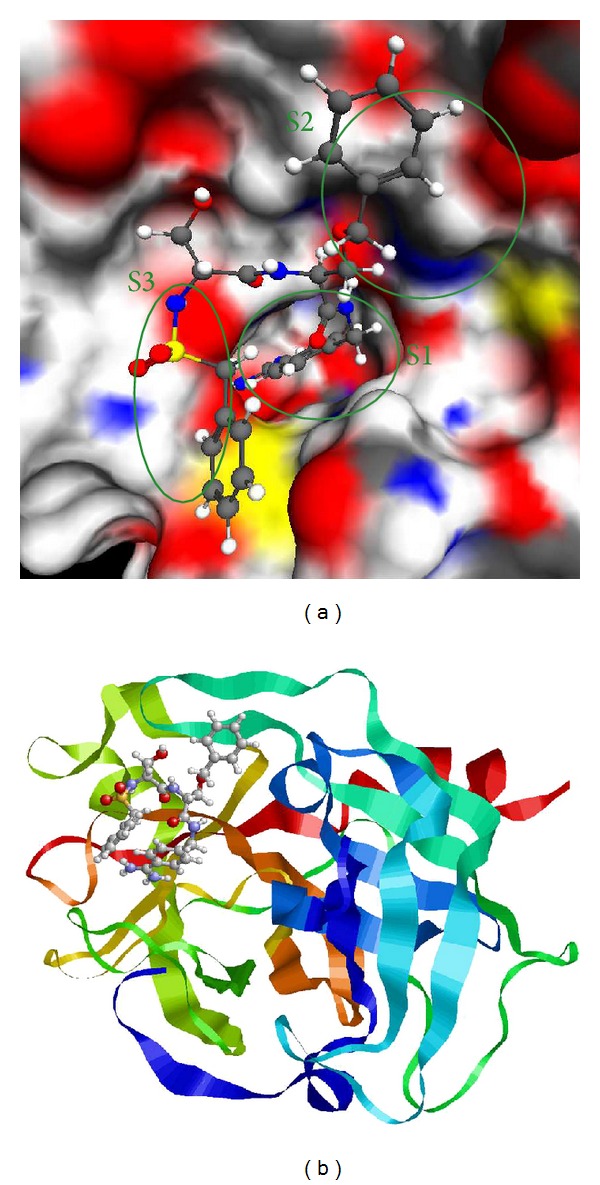
Complex 1VJ9 active site with location of the S1, S2, and S3 pockets indicated (program Molred) (a) and the full structure of the 1VJ9 complex (b).

Among these 60 structures, 45 ones contain uPA bound with direct reversible noncovalent inhibitors in its proteolytic active site. All these structures have rather good quality with resolution not worse than 3.1 Å. Most structures have resolution better than 2 Å. The structures of inhibitors are rather different representing different classes of chemical compounds, and there are no metal atoms nearby the active site. Three binding pockets can be distinguished in the proteolytic active site: S1, S2, and S3. Analysis of positions of uPA inhibitors crystallized in the complex with the protein demonstrates that the pocket S1 provides the main contribution to the binding, but the interactions with other pockets play also important role. The pockets and the full complex are demonstrated in Figure 1 for the crystal structure 1vj9 (PDB ID) from Protein Data Bank.

The availability of this data is very favorable factor for the rational drug design of the new uPA inhibitors using the molecular modeling methods. On the basis of existing crystal structures, the molecular model of the protein was build [33]. Crystal structures taken from PDB usually have no information about positions of the light atoms (hydrogens), so hydrogens were added to the protein structure before atom typification and grid building. This procedure was performed with APLITE program [34], which was also employed for the protein atom typification and the determination of the partial atomic charges in respect to MMFF94 force field [35–39]. Then, we build the potential grid in the predetermined docking area corresponding to the proteolytic active site of the uPA structure using SOLGRID program [34, 40, 41]. This area is a cube with the edge of 22 Å, indicating the rather large docking area which restricts the positioning space near the protein, but does not restrict the ligand movements in the active site which allows to find the energy global minimum using docking program. Overall, 45 uPA complexes crystallized with inhibitors were selected and prepared for docking. These structures were used as for docking quality testing, as for the further work with new inhibitors.

### 2.2. Modelling Methods

*Docking: The SOL Program.* Docking is currently the most common method of virtual screening. Docking is a ligand position search in a protein active site through global optimization (minimization) of the protein-ligand potential energy as a function of the ligand position. The ligand is a small molecule, which supposedly can inhibit the protein. As a result of docking, the ligand binding position and the protein-ligand binding free energy (which is correlated with the ligand inhibition ability) are predicted. We used the original SOL program [34, 40, 41] in this work. The SOL program finds global minimum of protein-ligand potential energy function by genetic algorithm. There are some assumptions in the SOL program to speed up calculations.

- The protein is considered to be rigid: there are no protein degrees of freedom in the potential energy function variables. But a broadening of the protein atomic potentials [40] with the typical value of 0.3-0.4 Å is used to take partially into account the protein atoms mobility.
- The ligand position search is performed inside the docking cube, covering the protein active site.
- The desolvation energy is calculated by simplified generalized Born model [42] and is included in the grid potentials.
- Energy of the protein-ligand interactions is calculated using the uniform space grid of the protein atoms potentials. This grid of the protein atoms potentials (Coulomb and Van der Waals potentials from MMFF94 force field with broadening and desolvation potential) is precalculated by SOLGRID program.
- There is no local energy optimization during the ligand position search.
- The ligand bond length and bond angles are kept fixed during the ligand position search; only torsion rotations around single acyclic bonds are allowed. Also, the ligand can be rotated and translated as a whole.
- Scoring function, which is an estimation of the protein-ligand binding free energy, is a weighted sum of the protein-ligand interactions energy components (Coulomb, Van der Waals and desolvation interactions) and entropy component, which is estimated by number of the ligand torsions. Coefficients in this sum have been adjusted to get best fitting of the calculated binding energy to experimental data.

The SOL program validation soon after its development [34, 43] has shown high docking quality: good rediscovery of a ligand native position for 80 protein-ligand complexes and good detection of active inhibitors among large set of inactive ligands by sorting by scoring function.

The SOL program was also tested in the Community Structure-Activity Resource (CSAR) competition in order to obtain an independent docking quality assessment. During this competition, the structures of proteins and ligands with unrevealed experimental native position and inhibition activity were given to all participants. Then, the participants tried to predict protein-ligand binding poses and sort ligands by their inhibitory activity. The SOL program demonstrated good ligand positioning quality (near resemblance of the predicted and native ligand poses) in most cases [34]: there were 6 proteins (Chk1, Erk2, LpxC, urokinase, CDK2, and CDK2-CYCLYNA) and 91 ligands in the competition, and 56 ligands of them were positioned by the SOL program with root mean square deviation (RMSD) over all the ligand atoms from the native position less than 2 Å and 65 ligands with RMSD less than 3 Å. It should be noted that there was no bad ligand positioning (with RMSD more than 3 Å) by the SOL program for LpxC, urokinase, CDK2, and CDK2-CICLINA proteins, while ligand positioning quality for Chk1 and Erk2 proteins by the SOL program was significantly worse.

Competition organizers assessed inhibition activity predictions by area under the curve (AUC) value. AUC is an area under the receiver operating characteristic (ROC) curve, which illustrates dependence of true positive rate on false positive rate, and thereby demonstrates quality of prediction method: AUC is equal to 1 in case of ideally accurate prediction and AUC is equal to 0.5 in case of the worst prediction. The SOL program shows high prediction quality of inhibition activity for LpxC protein (AUC = 0.95) and for urokinase (AUC = 0.97) and low prediction quality for Chk1 protein (AUC = 0.51).

The analysis of the SOL program results from the CSAR competition indicates that such a failure in ligand positioning and—as a consequence—failure in inhibition activity prediction may be the result of the inadequate protein flexibility model (potentials grid with broadening) and inaccurate solvent model. Nevertheless, the SOL program, despite its imperfection, took one of the first places in the CSAR 2011-CSAR 2012 competition [34].

### 2.3. Postprocessing: The DISCORE Program

As described above, there are some simplifications in the SOL program and there are false positive and false negative results of inhibition activity prediction, even in the cases of good ligand positioning. So, it is necessary to make protein-ligand potential energy function and scoring function more accurate. For this purpose, we developed postprocessing programs [34] to refine ligand positioning and, first of all, scoring from the SOL program through elimination of the SOL program simplifications. Scoring improvement during post-processing naturally involves two aspects:

1. ligand local optimization by L-BFGS [44] algorithm, starting from ligand binding position obtained from the SOL program, with taking into account solvent and explicit mobility of some protein atoms. Potential energy is calculated by MMFF94 force field without any simplifications. Solvation energy is calculated by one of three continuum solvent models (SGB, COSMO, or PCM) [45, 46]. This optimization, as well as the final binding energy component calculation is performed by the DISCORE program [34];
2. Scoring function coefficients adjusting by reliable experimental inhibition constants. This adjusting can be made specifically for the target-protein or for a generalized set of proteins.

Binding free energy (scoring function) is calculated by (1) in the postprocessing programs:

(1) ΔGbind=k1ΔGCoulomb+k2ΔGVdW+k3ΔGpol+k4ΔGnp+k5ΔGLS+k60.33NTORS+k7ΔGrot-tr,

where Δ*G*_Coulomb_ and Δ*G*_VdW_ are Coulomb and Van der Waals direct interaction energies in the frame of MMFF94 force field, Δ*G*_pol_ and Δ*G*_np_ are polar and nonpolar components of desolvation energy, Δ*G*_LS_ is the ligand strain energy, 0.33(kcal/mol∗torsion) × *N*_TORS_ is the entropy component caused by freezing of the ligand torsion degrees of freedom in binding process, *N*_TORS_ is the number of the ligand torsion degrees of freedom (number of single acyclic bonds, rotating heavy atoms), Δ*G*_rot-tr_ is the free energy component caused by loss of the ligand rotational and translational degrees of freedom in binding process [47], and *k*_*i*_  (*i* = 1, …, 7) are the unitless adjustment coefficients.

Coefficients *k*_*i*_ are adjusted by minimizing differences between calculated Δ*G*_bind_ and experimental binding energies (from articles or our experiments) determined using experimental inhibition constant by formula Δ*G* = *RT* × ln⁡(*K*_*i*_).

The GFIT program [34] is developed for this adjusting, which is made by iterative minimization of mean square differences between calculated Δ*G*_bind_ and experimental binding energies.

We also have the possibility to show the postprocessig results (not only docking results) during the CSAR competition [34] to check the quality of the DISCORE program. So, the DISCORE program results were also presented at the competition and compared with the results of other groups. Unfortunately, inhibition activity prediction by the DISCORE program has improved prediction by the SOL program, not for all proteins. Ignoring cases with bad initial positioning by the SOL program (so improvement of this positioning and scoring could not be made by the DISCORE program), there were designated two major reasons of low postprocessing quality: poor solvent model parameterization and very simple empiric entropy calculation (by counting of the ligand torsions) both in the SOL and DISCORE programs.

### 2.4. Quantum Chemistry

Quantum chemistry methods are another important molecular modeling tool. Strictly speaking, exact calculation of intermolecular interactions can be done only with quantum chemistry, so quantum chemistry application in the drug design can significantly improve accuracy of calculations. But quantum chemical calculations require a lot of memory (both RAM and HDD) and a lot of CPU time: a system with tens atoms can be being processed within ten hours at one CPU. So,* ab initio* quantum chemistry methods cannot be applied to proteins. But semiempirical quantum chemistry methods are not so resource-demanding. In this work, we used the MOPAC package of semiempirical programs [48] developed by Stewart. Today, it is one of the fastest and most undemanding quantum chemical programs. Traditional quantum chemistry methods, including semiempirical methods, use some matrix algebra methods with complexity of *O*(*N* ^3^), where *N* is a number of atoms, so it is impossible to process system of more than 1000 atoms by these methods. But with the implemented MOPAC package localized molecular orbitals (LMO) method, equations of self-consistent field can be solved by *O*(*N*) operations. Also, there are speedups of other resource-demanding stages of calculations, so the MOZYME module [49] of the MOPAC package allows processing a system of 15 000 atoms, including proteins and protein-ligand complexes.

In this work, we calculated protein-ligand binding energies for urokinase by the PM7 [50] parameterization method, implemented in the MOPAC package. Corrections for intermolecular interactions (dispersion interactions, hydrogen bonds, and interactions with halogens) [51–54] were taken into account in the PM6-DH2X and PM7 parameterizations. Before binding energy calculation, all ligand atoms and some protein atoms (about 10–15 amino acids of the active site) were locally optimized with L-BFGS method, implemented in the MOPAC package. Solvent (COSMO model) was taken into account only for binding energy calculation, not during optimization. Semiempirical methods, despite of all their speed, were lost in accuracy* ab initio* methods for a long time, because of their poor nonvalence interactions modeling, particularly, dispersion interactions and hydrogen bonds. And underestimation of repulsion forces leads to overstating of interaction energies and, as consequence, decreasing of distances between atoms in the equilibrium state. However, additional empirical corrections are solving this problem. As a result, the PM6 parameterization method with hydrogen interaction corrections has accuracy better than 1 kcal/mol (by interaction energies in the test set S22 including 22 molecules [51]). The PM6 method with dispersion interactions corrections and interactions with halogens corrections differs from* ab initio* methods by less than 10% in energy calculations of interactions with halogens. Semiempirical methods with all these corrections can reach accuracy of the DFT-D (DFT including semiempirical dispersion interactions) methods in most cases, but these semiempirical calculations can be done faster by three orders [53].

All of these corrections are included into the latest parameterization method in the MOPAC package, PM7 method, which is modified PM6 method. The PM7 parameterization is based on experimental and high-level* ab initio* data. Also in the PM7 parameterization method, two insignificant mistakes are fixed, which appear in large system processing. This has significantly improved the accuracy of large organic systems and solids calculations.

### 2.5. Generalized Direct Docking: The FLM Program

Described above methods use only one ligand position in the protein active site (the position of the potential energy global minimum) to calculate protein-ligand binding energy. But a ligand in thermodynamic equilibrium state can be continuously transforming from one binding pose to another due to the thermal motion of its atoms. Binding is not a static event, but a dynamic process [55–58]. Thus, protein-ligand binding free energy calculation can be improved with taking into account multiple poses both of a bound ligand and of a free ligand. Besides, the protein-ligand binding constant depends not only on potential energy but on free energy, consisting of enthalpy and entropy, which can be calculated more realistically through ligand movement accounting.

Free energy *G* can be derived from statistical sum *Z* by (2) [59]:

(2) $$\begin{array}{c} G=-kT\ln\left(Z\right). \end{array}$$

Low-energy states make major contribution to the statistical sum, so not only the global energy minimum, but also local energy minima, close to the global minimum in terms of energy, and contribute to the free energy. The Find Local Minima (FLM) program of direct gridless docking was developed on the basis of this assumption. The FLM program searches local minima of the protein-ligand complex and the free ligand by the Monte-Carlo method: random torsion deformations and random rotations-translations are applied to the ligand, and then ligand is locally optimized by the L-BFGS algorithm. Performance of a lot of such independent local optimizations results in determination of a set of local minima. Up to 1024 different local minima with the lowest potential energies are being kept in this set. After the local minima search is completed, each of the local minima is approximated by harmonic oscillator (approximation based on the potential energy hessian), and its natural frequencies are calculated. So, statistical sum *Z* ^*i*^ over the *i*th local minimum configurational space and corresponding free energy *G* ^*i*^ can be calculated by (3) as follows:

(3) Gi=−kTln⁡(Zi)=−kTln⁡(e−E0i/kT∗Zvi∗Zti∗Zri)=E0i+Gvi+Gti+Gri,

where *E*_0_^*i*^: potential energy of the *i*th local minimum, *Z*_*v*_^*i*^, *Z*_*t*_^*i*^, and *Z*_*r*_^*i*^: statistical sum components over the *i*th local minimum configurational space associated with the vibrational [60], translational, and rotational [47] degrees of freedom respectively, and *G*_*v*_^*i*^, *G*_*t*_^*i*^, and *G*_*r*_^*i*^—corresponding free energy components.

Then, the total statistical sum *Z* and corresponding free energy *G* can be calculated with taking into account many local minima by (4) as follows:

(4) $$\begin{array}{c} G=-kT\ln\left(Z\right)=-kT\ln\left(\sum_{i}Z^{i}\right)=-kT\ln\left(\sum_{i}e^{-G^{i}/kT}\right). \end{array}$$

We named this approximation as “multiwell approximation.”

One of the FLM program features is the absence of adjusting coefficients: all energy calculations are performed directly by the MMFF94 force field, when most of the docking programs calculate scoring function with nonphysical coefficients or even not in energy units.

In this work, the FLM program was used as a next step of the docking improvement and of the binding energy calculation improvement. We named docking procedure in the FLM program as a “*gridless*,* direct*,* generalized*” docking by these reasons: docking is “*gridless*” because the protein potentials grid is not used (unlike the SOL program and most of other docking programs); docking is “*direct*” because all energy calculations were performed directly in the MMFF94 force field without any adjusting parameters; and docking is “*generalized*” because many low-energy local minima, not only the one global energy minimum, are found and processed during calculations. Also, the FLM program searches for minima of the free ligand, not only for minima of the protein-ligand complex, unlike what most of the docking programs do. This allows to take into account the ligand deformation energy (or the ligand strain energy)—energy of the ligand deformation from its free conformation to its bound conformation—which is included to the protein-ligand binding energy. Our computations show that this deformation energy can vary from a few to tens of kcal/mol, so its accounting is important for binding energy calculations.

### 2.6. Experimental Technique of Urokinase Inhibition Testing* In Vitro*

After selection of the most promising candidates for new urokinase inhibitors, we tested their inhibition activities* in vitro*. The experiments were carried out on the system with the urokinase specimen, the tested ligand, and the special substrate—a synthetic Pyro-Glu-Gly-Arg-pNA peptide (S-2444) [61, 62]. Urokinase decomposes the substrate with production of the chromogenic product. When the inhibitor binds to the urokinase active site, this production is slowed down. The higher the inhibition activity of the ligand, the slower the working of urokinase and the slower the accumulation of chromogenic product. Thus, the inhibitory activity can be measured by the rate of optical density change at the 405 nm wavelength. The suggested method of the inhibitory activity measurement is suitable to obtain inhibition percentage, EC_50_, IC_50_. Error of the method for IC_50_ measurement in micromole range is about 7–10 *μ*M.

## 3. Results and Discussion

### 3.1. Docking Results of the Native Protein-Ligand System: Docking Quality Validation

The validation of the docking program quality is a way to estimate whether it positions native ligands correctly into the active site of the target-protein as well as to evaluate accuracy of the protein-ligand binding free energy calculation. The correct docking program enables to find true inhibitors among a large number of inactive compounds. The validated program is the docking program SOL. The native ligands (for particular target-proteins) are the ligands whose binding poses in the active site of the target-protein are known from experiments.

The quality positioning is defined by root mean square deviation (RMSD) between all atoms of native ligand poses and docked ligand poses. We calculated 45 native protein-ligand complexes taken from Protein Data Bank. The commonly assumed docking quality gradation is “excellent” in the case of RMSD < 1 Å, “good” in the case of 1 Å < RMSD < 2 Å, “satisfactory” in the case of 2 Å < RMSD < 3 Å, and “bad” in the case of 3 Å < RMSD. Distribution of the native ligand docking results (absolute and relative quantities) by quality is shown in Table 1. As it can be seen from Table 1, most of the native ligand docking results (80%) have “satisfactory” or better quality.

The quality of the urokinase-ligand binding free energy prediction was also tested by comparing predicted energies with the experimental data. We have found urokinase inhibition constants *K*_*i*_ for all 45 native ligands from various literature sources, and then we transformed these inhibition constants to the binding free energies by formula Δ*G* = *RT* × ln⁡(*K*_*i*_). Then, the predicted binding energies were correlated with the experimental binding energies. In addition to the continuous energy values correlation, we also tested correlation of a binary classification (“inhibitor”-“noninhibitor”) between calculated and experimental results. A ligand is classified as “inhibitor” in the case of *K*_*i*_ < 10 *μ*M for the experimental data and in the case of SCORE < −5.5 kcal/mol for the calculated data. Correspondingly, a ligand with *K*_*i*_ > 10 *μ*M is classified as an experimental “non-inhibitor,” and a ligand with SCORE > −5.5 kcal/mol is classified as a predicted “non-inhibitor.” A mismatch between experimental and calculated classification is called “false positive” (FP, when ligand is an experimental “non-inhibitor,” but predicted as “inhibitor”) or “false negative” (FN, when ligand is an experimental “inhibitor,” but predicted as “non-inhibitor”). The numbers of such mismatches and the continuous energies correlation coefficient for the tested ligands are shown in Table 2.

For the conjectural inhibitors selection from a big database and their subsequent experimental synthesis and verification, first of all, it is necessary to decrease a “false positive” number. We tried to use postprocessing procedure to improve prediction quality and decrease numbers of “false positive” and “false negative” results.

Another way to estimate docking quality, with focus on ability of the active inhibitors selection, is the enrichment plot (*E*-plot) and enrichment value (*S*_*E*_) [63], where the latter is the area under the* E*-plot. This plot represents the relative number of known, real inhibitors (normalized by the total number of known, real inhibitors found in the whole set of ligands under consideration) plotted as a function of the number of top-scoring compounds needed to include those inhibitors. For example, if 8 (known) real inhibitors are found among the 2000 test compounds and the top 200 of these compounds include 4 real inhibitors, then the corresponding* E*-plot point has coordinates in percent of (*x* = 10, *y* = 50) because 200/2000 = 0.10 and 4/8 = 0.50. The area *S*_*E*_ below* E*-plots can be used to quantify the observed enrichment of real inhibitors. Enrichment values *S*_*E*_ greater than 0.9 are excellent, and enrichment values *S*_*E*_ below 0.6 represent no enrichment [63].

The* E*-plot can be obtained by the next algorithm: (1) list of all ligands is sorted by scoring function; (2) first *N* ligands are selected from this list, and *K* ligands of them are active inhibitors; (3) the point (*N*/*N*_all_, *K*/*K*_all_) is plotted, where *N*_all_ is the total ligands number in the dataset and *K*_all_ is the total active inhibitors number in the dataset. Steps (2) and (3) are repeated with *N* varied from* 0* to *N*_all_. The closer the *S*_*E*_ to value 1, the better quality of the docking program.

**Figure 2:**
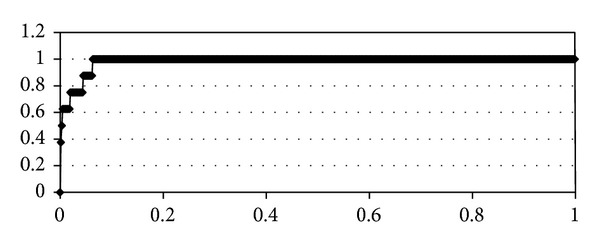
*Enrichment plot* by the SOL docking program: the 7 active urokinase inhibitors are selected from the 1888 assumed to be inactive ligands of the NCI Diversity database.* Enrichment value* is equal to 0.98.

The* E*-plot for the 7 active urokinase inhibitors (*amiloride *[64]*, B428 *[20]*, uk122 *[21–31]*, wx-uk1* [21–31]*, wx-293-t* [21–31]*, wx671* [21–31]*, and B623* [20]) selection from the 1888 ligands of the NCI Diversity database [65] by the SOL program score is shown in Figure 2. All 1888 ligands of the NCI Diversity database have been assumed to be inactive.

The docking program with such a high *S*_*E*_ of 0.98 is considered to be very effective for the active inhibitors selection from a large set of inactive ligands. It should be noted, however, that there can be other unknown urokinase inhibitors among the 1888 ligands of the NCI Diversity database, because these ligands have not been experimentally tested for urokinase inhibition: the true *S*_*E*_ can differ from specified value of 0.98. So, the SOL docking program has both good quality of ligand positioning in the urokinase active site and good quality of separation active urokinase inhibitors from inactive.

Also, we estimated influence of the urokinase structure on the docking results by crossdocking procedure—the docking of the ligand from one PDB complex into the protein from another PDB complex. In other words, crossdocking procedure demonstrates effect of the protein flexibility on the protein-ligand binding process. Score of the protein-ligand binding for the protein and ligand of the same PDB complex varies within the average of 1 kcal/mol with the protein structure change. It indicates some urokinase flexibility in the binding process, but this effect can be neglected.

Then, we have chosen the urokinase structure (1SQO protein from the PDB [66]) for subsequent calculations: docking by the SOL program, postprocessing by the DISCORE program, quantum chemistry calculations by the MOPAC package, and generalized docking by the FLM program.

### 3.2. Docking: Virtual Screening Databases Containing Ready Compounds

Search of the uPA inhibitors in ready-made compounds databases is the firststage of the new inhibitor development. The technique of virtualscreening can be summarized as the following: we choose the database of drug-like compounds, which have been already studied as potential drugs with respect to some other target-proteins and have been already synthesized, so small amounts of these compounds can be ordered for experimental tests either free of charge or at small price. Then, we perform docking of the compounds from the database into a given target-protein and select, if any, the candidate inhibitors, which are ordered and tested in experiment. If inhibitory activity of compound is confirmed in experimental tests, it serves as a basis for design of new patented inhibitors. In addition to experimental confirmation of inhibitory activity predictions by docking programs and to validation of the experimental test systems, this procedure makes it possible not only to find out new molecular groups, which form inhibitors and play an important role in binding with target-protein, but also to discover a new application of the known compounds.

In our research, we have started from ZINC [67] database, which is the library of the compounds prepared for docking and provided by various suppliers from all over the world. About 800 thousands of compounds from the “lead-compound” set (small compounds which can be used as a basis to construct more heavy-weighted inhibitors) were docked. Calculations were carried out using parallel mode of “LOMONOSOV” supercomputer (MSU). Then, we ranged compounds by scoring function and analyze the ones which are in top positions and have scoring functions <−5,5 kcal/mol. We explore the potential for order taking into account our facilities to order compounds and came in contact with Russian databases, such as ACB-Blocks [68] and Vitas-M [69], and 43 compounds have been ordered. Also, two compounds were obtained from our colleagues and one compound was obtained from Alfa Aesar database. All these compounds were tested experimentally, and 14 compounds displayed some inhibitory activity (IC_50_~200 *μ*M is the best result). These both positive and negative results were used during the next stages of the new uPA inhibitors development. We also perform docking of 1888 compounds from NCI Diversity [65] and expect that large ligands will give better results in experimental tests than small ligands with the same scoring functions. However, the selected compounds were not ordered because of technical problems. Those 23 compounds which are in top positions by SOL scoring function are presented in Table S2 in Supplementary Material available online at http://dx.doi.org/10.1155/2014/625176.

### 3.3. Postprocessing

In order to carry out training and testing of the postprocessing program, we divide 88 available compounds (45 native compounds and 43 compounds ordered from databases) into two sets: training set (50 compounds) and test set (38 compounds) in the way that there are both active and inactive ligands in each set. Then, the adjustment coefficients for energy components of the scoring function were determined using the GFIT program (Table 3), and the corrected scoring function values were calculated for two sets accounting these adjustment coefficients. On the basis of these calculations, numbers of false positives and false negative were estimated again, and the correlation coefficient with experimental data was also calculated (Table 4).

It can be seen from Table 4 that the postprocessing application increases significantly the correlation coefficient between calculated and experimentally measured protein-ligand binding energies and decreases appreciably number of false positives predictions.

A next step was to train the DISCORE program by total of 88 available uPA-ligand complexes to perform further search of the new inhibitors (Tables 5 and 6).

Number of false positives decreases more in this case while the correlation coefficient between calculated and experimental data is still rather high. Clearly, if the compounds, which are selected on the basis of docking results, are to be synthesized, then we should strive for the least number of false positive ligands. The postprocessing DISCORE program and the adjustment of the appropriate coefficients help to decrease empirically the number of false positives in this case.

According to the calculations performed using the DISCORE program, we also can suggest that low correlation coefficient between experimental and calculated energies obtained using the SOL program may result, first, from insufficiently accurate accounting of entropic component of binding free energy in SOL scoring function, second, from the simplified accounting of the solvation energy and, thirdly, from the absence of the ligand local optimization into the found global minimum.

### 3.4. MOPAC

MOPAC software makes it possible to perform the local optimization over the selected atoms of the molecular system, so at the first of calculations there was an attempt to optimize not only ligand in the protein active site but also the protein amino acids that are nearest to it. 10–15 such amino acids were chosen, that increased the calculation time more than twice, but the final binding energies only slightly changed (by an average of 5%), that also did not affect the correlation with the experimental data. So, the decision was made to perform the optimization over the ligand atoms only.

We also consider the versions of the optimization performing in solvent (COSMO) and in vacuum. It was demonstrated that the optimization in solvent did not improve the final results but increases significantly the calculation time, so the decision was made to account the solvent at the last optimization step only.

The above calculation parameters were tested using 8 native urokinase-ligand complexes from the PDB Database, chosen so that there were inhibitors of different size and activity among them. The correlation coefficient between binding energies obtained by MOPAC and experimental binding energies is 0.68 (for 8 tested complexes), but any additional adjustable parameters have not been used. The correlation coefficient between binding energies obtained by SOL and experimental binding energies is 0.6 for the same complexes, but there are some empirically chosen parameters in the SOL score. The additional calculations by MOPAC using both active and inactive ligands enabled us to choose threshold enthalpy value, which makes it possible to separate the compounds potentially active. For urokinase ligands, this value equals −40 kcal/mol.

This suggests that including quantum chemistry into the binding free energy computing process can improve the precision of the calculations without using the additional adjustable parameters, which are presented in the scoring-functions of the SOL program as well as of the DISCORE program. However, since time required for the calculation of one complex varies from one to ten hours, the quantum chemical virtual screening of databases containing tens and hundreds of thousands of compounds is still unrealizable now.

### 3.5. FLM and the Multiwell Approximation

The set of 8 urokinase-ligand native complexes from the PDB Database for the FLM program calculations has been chosen. The time required to perform the calculations depends on the size of the ligand. It takes less than 1000 CPU∗hours to find the minimum nearby the global minimum for the simple ligands of the size which is not more than 5 torsions. We consider that the global minimum is found when the pool of minima with the lowest energies stops accepting new local minima. However, for the large ligands (about 17 torsions), the calculation lasts about 20000 CPU∗hours without attainment of the selected local minima saturation.

The local minimum with the lowest energy is also close to the native ligand position (RMSD < 3 Å) for 6 complexes from 8. For the other 2 complexes program finds the minimum nearby the native ligand position by RMSD, which also has low energy, but which is different from the global minimum. Generally speaking, the native ligand position is not forced to correspond to the global minimum of the protein-ligand system potential energy. Several factors can influence on such result: inadequate description of the protein-ligand interaction energy in the model system (e.g., in our case minima search was performed without solvent), inadequate description of the intermolecular interactions by the force field used, or the random perturbations during the complex crystallization process.

Further, potential binding energy was computed using the FLM program, and by means of this energy enthalpy, entropy and binding free energy were determined. Calculations were carried out without solvent, and the correlation coefficient between calculated energies and experimental values was 0.5.

As it was mentioned above, the quantum chemical calculations can be perspective to find accurate protein-ligand binding energies. So, we undertook the following step: we performed enthalpy calculations by MOPAC for the global minimum found using FLM in two variants using local optimization of the global minimum of the ligand in the protein and without local optimization. Correlation coefficients with experimental values are 0.46 and 0.44, respectively. However, it should be taken into account that in this case the enthalpy calculation using MOPAC is only performed for only one minimum with the lowest energy, but as it was mentioned before the lowest energy position has RMSD > 3 Å from the native ligand position in the protein for 2 ligands from 8. Entropic contribution of the binding free energy was not also taken into account in this case. These two features can negatively affect the correlation between MOPAC enthalpies and experimentally determined binding free energies.

### 3.6. Rational Design and Experimental Testing of New uPA Inhibitors

All ordered compounds (50 compounds) were tested experimentally* in vitro* using procedures described in “Methods.” Compounds which display activity, their IC_50_ values, and also calculated binding energies using programs SOL, DISCORE, and MOPAC are presented in Table S2 in Supplementary Materials, and the some characteristic representatives are presented in Table 7.

Besides the validation of experimental activity for compounds, selected by virtual screening calculations, we also suggested several scaffold-like molecules for the synthesis as the potential uPA inhibitors. The structures of synthesized compounds are presented in Table 8 together with binding scores calculated using programs SOL, DISCORE, and MOPAC. These scores were used separately, and the “consensus” score was not used, because we found that MOPAC score predicted activities much better than MMFF94 force field, and there is no sense to combine it with SOL and DISCORE scores. However, we cannot abandon the latter two scores, because they are available in the virtual screening of many compounds.

All these compounds contain the positive-charged fragment (amine, guanidine, and thiouronium), which can interact with Asp189 residue in the uPA active site.

There are several points, which we wish to address by the validation of these synthetic structures in experiment. First of all, it was interesting to substitute the guanidinium charged group by thiouronium fragment in well-known structure of guanidinium-containing uPA inhibitors [23, 64]. This substitution seems to work well in design of thrombin inhibitors [70], but, from Table 8, it is evident (compounds 1–3) that, in the case of uPA, the thiouronium-containing compounds turned out to be completely inactive, whereas their guanidinium counterparts have significant activity [23, 64]. Because thrombin and uPA have similarity in their active sites structures, the reason of such behavior remains unclear.

The next set of inhibitors (compounds 4–15 in Table 8) can be considered as modifications of known structures of uPA inhibitors: 2-aminobenzimidazole and 2-aminoquinoline [64, 71]. The docking experiments verified that such modifications can provide the reasonable fit of selected compounds with uPA active site. Despite this promises, all the synthesized compounds are of little interest as uPA inhibitors. An additional activity was performed by synthesizing the compounds 16–18 (Table 8), which can be regarded as simplified and “cyclized” amiloride [64] structures. In this case, the compounds display substantial activity, especially the sulfur-containing substituted 1,2-benzisothiazoles. The favoring influence of sulfur may be due to the enhanced Van der Waals interaction with the uPA active site. The insertion of bromine into position 5 of 1,2-benzisothiazole ring (which corresponds sterically to chlorine position in amiloride molecule) also enhances the activity, signifying the importance of bulk electron deficient substituent at this site. Finally, the 3-guanidino-1,2-benzisothiazole can be regarded as new patent-free scaffold, which is the good starting point for subsequent design of new uPA inhibitors.

**Figure 3:**
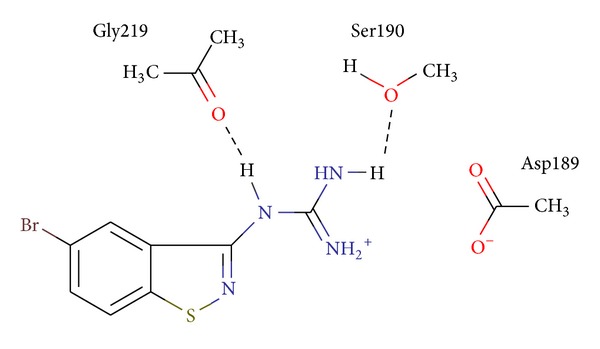
Specific interactions of the U026R with the aminoacids of the urokinase binding site.

As a result, there are 8 active compounds between 18 synthesized compounds, though their activities are generally weak. IC_50_ of six compounds is equal to 200 *μ*M and more. Among substances, which we synthesized for urokinase inhibition tests, the two most active were 1,2-benzisothiazol-3-ylguanidine (IC50 = 33 *μ*M) and its 5-bromo derivative (4–20 *μ*M). The specific interactions of the U026R with the aminoacids of the urokinase binding site are shown in Figure 3. The electrostatic interaction with the Asp189 and the formation of the hydrogen bonds with Gly219 and Ser 190 can be marked out.

**Scheme 1:**
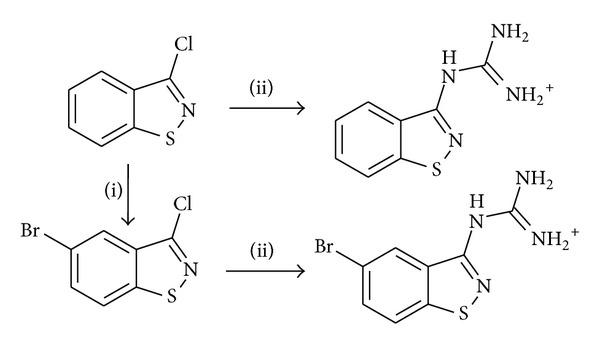
A synthesis scheme of 1,2-benzisothiazol-3-yl-guanidine (U024R) and (5-bromo-1,2-benzisothiazol-3-yl) guanidine (U026R).

They were obtained from the corresponding chlorides via prolonged heating with excess ethanolic guanidine. (A synthesis scheme is presented in Scheme 1 and in Supplementary materials). It should be noted, however, that all these compounds including the most active ones are small structures, which can be enlarged by addition of new substitutes, which may affect the activity either positively or negatively.

**Figure 4:**
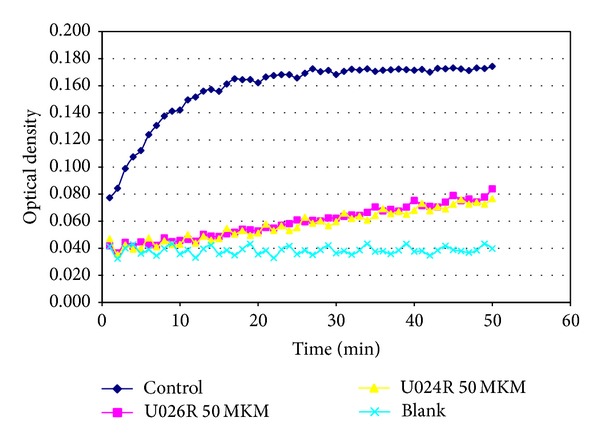
Inhibition of uPA proteolytic activity by U024R and U026R (50 *μ*M) was measured by decomposition of the specific chromogenic substrate S-2444.

Experimental tests were performed as indicated in paragraph 3.5 of “Methods.” Urokinase proteolytic activity in the presence of inhibitor (and also in absence of inhibitor, labeled “control” in Figure 4) was determined by releasing the paranitroanilide by means of measuring of absorption with a wave length of 405 nm each minute at 30°C. Figure 4 is a diagram of relationship between optical density and time. The angle of curve describes the reaction rate: the lower it is (compared with “control”, which corresponds to urokinase without inhibitors), the better inhibitor works.

## 4. Conclusions

This work focuses particularly on the initial rational drug design process, development of new inhibitors the targeted the specified proteins using the molecular modeling techniques and supercomputer-based calculations: basis of the target-protein choice, building of the molecular model, screening of ready-made compounds databases using docking and postprocessing programs, and selection of the best inhibitor candidates based on the calculations results and checking their correspondence to experimental data, new compounds design, their synthesis, and experimental testing.

Particular effort was made to investigate different docking methods and protein-ligand binding energy computation methods. Additional researches have discovered that the potential binding energies of the free protein, free ligand, and their complex global minima are the key values when comparing binding energies of different ligand to select the best or the worst inhibitor, while accounting many local minima near the global one and molecules oscillations in these minima play the role of correction for the potential energies. Accounting of rotations and translations leads all energies to change by about the same amount, so it also plays the role of a small correction when comparing binding free energies of two ligands.

It was shown that the correlation between theory and experiment can be increased using the recently developed quantum chemical semiempirical parameterization method PM7 focusing on the description of the intermolecular interactions that is more accurate than in previous semiempirical methods.

Further development is likely associated with more consistent and accurate accounting of solvent, with more regular global minimum search and nearby local minima search, with more accurate potential energy calculations: using quantum chemical methods or other alternative to MMFF94 force field, and with accounting the mobility of atoms in the protein active site.

Since development and application of the previously mentioned programs for computer modeling were carried out simultaneously with the new inhibitors search, the calculation results for some compounds have been obtained after their synthesis and experimental tests, and thus it became additional quality estimation criterion for these programs. So, for example, the postprocessing gave quite good results for native ligands at the first stage of development, but it predicts activity worse than SOL docking program for the synthesized ligands. Probably this is associated with defects of MMFF94 force field, with too rough calculations of entropy contribution, and also with using of empirical adjustment coefficients.

In contrast to the postprocessing based on MMFF94 force field, the quantum chemical calculations have shown better results than the docking program SOL. The correlation between experimental data and energies computed by MOPAC for our synthesized ligands is 0.52. So, we can put forward the suggestion that employment of the semiempirical quantum chemistry PM7 method with docking allows not only to divide ligands into “bad” and “good” but also to separate “good” inhibitors from “satisfactory” ligands and “very good” ligands.

As a result of the performed research, 18 novel compounds were designed, synthesized, and tested in experiments, and two of them have demonstrated inhibiting activity ~10 *μ*M. These compounds can be the basis for the further drug development and for obtaining the uPA leader inhibitors, which are perspective in terms of design of the new chemical class of antitumor drugs. This requires not only to increase new inhibitors activity at least by an order of magnitude but also to improve significantly the accuracy of* in vitro* inhibitory activity measurements.

## Supplementary Material

Supplementary materials present the structures and docking score results of the compounds selected from NCI Diversity Database. These results are summarized in table S1, which contains NCI ID of compounds, their chemical structures, SOL Score and enthalpies calculated by MOPAC. Also supplementary materials include the table S2, containing chemical structures, docking (SOL Score), postprocessing (DISCORE Score) and FLM (FLM Score) score results, MOPAC enthalpies and experimental uPA inhibition activity (IC_50_) of compounds, which were ordered from ACB-Blocks and Vitas-M databases of ready compounds. Supplementary materials also contain the description of synthesis of (5-bromo-1,2-benzisothiazol-3-yl)guanidine hydrochloride and duplicate the table 8, which include chemical structures, docking (SOL Score), postprocessing (DISCORE Score) and FLM (FLM Score) score results, MOPAC enthalpies and experimental uPA inhibition activities (IC_50_) of synthesized compounds.

## Figures and Tables

**Table 1:** The native ligand positioning quality by the SOL program for urokinase protein.

RMSD range	Number of the native ligands	Percentage of the native ligands
RMSD > 3 Å	9	20%
RMSD < 3 Å	36	80%
RMSD < 2 Å	21	47%
RMSD < 1 Å	5	11%

**Table 2:** Correlation coefficient between predicted and experimental binding energy values and numbers of “false positive” and “false negative” results. Predicted results are calculated by the SOL docking program for the 45 native ligand-urokinase complexes.

Correlation coefficient	0.35	
“false positive” number	2	Total of 9 mismatches out of 45 results
“false negative” number	7	

**Table 3:** Adjustment coefficients of formula (1), determined using the GFIT program GFIT and the training set of 50 uPA-ligand complexes.

*k*_1_	*k*_2_	*k*_3_	*k*_4_	*k*_5_	*k*_6_	*k*_7_
0.0292	0.1295	0.2437	−0.0438	0.4958	0.2597	0.0393

**Table 4:** Comparison of correlation coefficients and numbers of false positives and false negative results (a total of 88 complexes) for the SOL program and the DISCORE program trained by 50 uPA-ligand complexes.

	Correlation coefficient	Number of false positives	Number of false negative
SOL docking	0.35	26 of 88	7 of 88
DISCORE postprocessing (training set)	0.58	8 of 50	3 of 50
DISCORE postprocessing (test set)	0.52	9 of 38	4 of 38
DISCORE postprocessing (all complexes)	0.55	17 of 88	7 of 88

**Table 5:** Adjustment coefficients of formula (1), determined using the GFIT program GFIT and the training set of 88 uPA-ligand complexes.

*k*_1_	*k*_2_	*k*_3_	*k*_4_	*k*_5_	*k*_6_	*k*_7_
0.0387	0.1387	0.1162	−0.0188	0.1081	0.0504	0.0322

**Table 6:** Comparison of correlation coefficients and numbers of false positives and false negative results (a total of 88 complexes) for the SOL program and the DISCORE program trained by 88 uPA-ligand complexes.

	Correlation coefficient	Number of false positives	Number of false negative
SOL docking	0.35	26 of 88	7 of 88
DISCORE postprocessing (training set)	0.59	10 of 88	8 of 88

**Table 7:** Structures, docking, and postprocessing score results, MOPAC enthalpies, and experimental uPA inhibition activity (IC_50_) of ordered compounds.

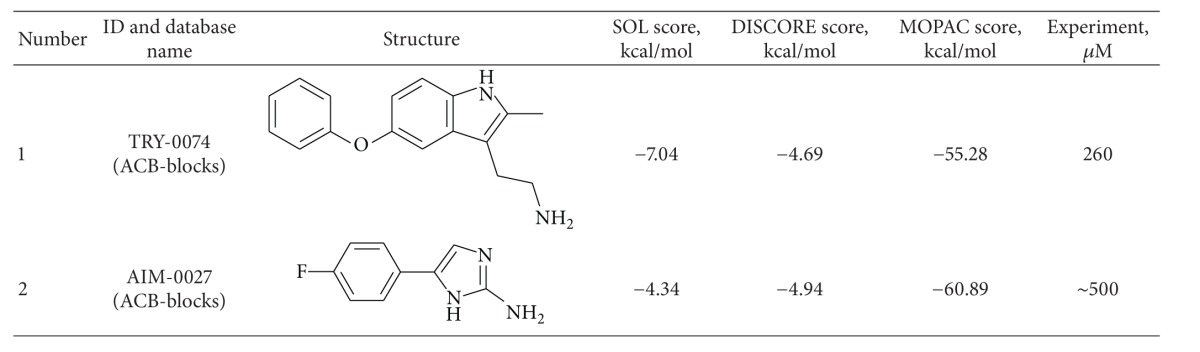

**Table 8:** Structures, docking, and postprocessing score results, MOPAC enthalpies and experimental uPA inhibition activity (IC_50_) of synthesized compounds. N.b.: non binder.