# Supplementary material for: Application of Molecular Modeling to Urokinase Inhibitors Development
Source: Biomed Res Int. 2014 May 20;2014:625176. doi: 10.1155/2014/625176 (PMC4055159; doi:10.1155/2014/625176)
Supplement: Supplementary file 1 — Supplementary materials present the structures and docking score results of the compounds selected from NCI Diversity Database. These results are summarized in table S1, which contains NCI ID of compounds, their chemical structures, SOL Score and enthalpies calculated by MOPAC. Also supplementary materials include the table S2, containing chemical structures, docking (SOL Score), postprocessing (DISCORE Score) and FLM (FLM Score) score results, MOPAC enthalpies and experimental uPA inhibition activity (IC50) of compounds, which were ordered from ACB-Blocks and Vitas-M databases of ready compounds. Supplementary materials also contain the description of synthesis of (5-bromo-1,2-benzisothiazol-3-yl)guanidine hydrochloride and duplicate the table 8, which include chemical structures, docking (SOL Score), postprocessing (DISCORE Score) and FLM (FLM Score) score results, MOPAC enthalpies and experimental uPA inhibition activities (IC50) of synthesized compounds. [file 625176.f1.doc]

**Supplementary materials**

**Table S1.** Structures and docking score results of selected NCI compounds.

| № | NCI ID | Structure | SOL Score, kcal/mol |
| --- | --- | --- | --- |
| 1 | NSC77554 |  | -8,23 |
| 2 | **NSC639174** |  | -7,52 |
| 3 | **NSC74472** |  | -7,28 |
| 4 | **NSC347197** |  | -7,21 |
| 5 | **NSC357777** |  | -7,09 |
| 6 | **NSC190336** |  | -7,02 |
| 7 | **NSC13545** |  | -6,93 |
| 8 | **NSC118396** |  | -6,83 |
| 9 | **NSC89110** |  | -6,82 |
| 10 | **NSC154966** |  | -6,70 |
| 11 | **NSC18355** |  | -6,69 |
| 12 | **NSC153533** |  | -6,626 |
| 13 | **NSC326381** |  | -6,49 |
| 14 | **NSC26904** |  | -6,49 |
| 15 | **NSC14288** |  | -6,48 |
| 16 | **NSC62857** |  | -6,44 |
| 17 | **NSC117369** |  | -6,34 |
| 18 | **NSC52075** |  | -6,30 |
| 19 | **NSC35605** |  | -6,24 |
| 20 | **NSC53069** |  | -6,19 |
| 21 | **NSC305831** |  | -6,16 |
| 22 | **NSC****143125** |  | -6,08 |
| 23 | **NSC528168** |  | -6,57 |

**Table S2.** Structures, docking and postprocessing score results, MOPAC enthalpies and experimental uPA inhibition activity (IC_50_) of ordered compounds.

| № | ID and Database Name | Structure | SOL Score, kcal/mo | DISCORE Score, kcal/mol | MOPAC Score, kcal/mo | Experiment, μM |
| --- | --- | --- | --- | --- | --- | --- |
| 1 | TRY-0003 (ACB-Blocks) |  | -6,29 | -3,91 | -49,79 | 500 |
| 2 | AIM-0027  (ACB-Blocks) |  | -4,34 | -4,94 | -60,89 | 500 |
| 3 | AIM-0007  (ACB-Blocks) |  | -4,73 | -5,01 | -59,75 | 500 |
| 4 | TRY-0004 (ACB-Blocks) |  | -7,1 | -4,54 | -55,26 | >500 |
| 5 | TRY-0023  (ACB-Blocks) |  | -7,47 | -4,85 | -51,86 | 420 |
| 6 | TRY-0025  (ACB-Blocks) |  | -7,42 | -4,64 | -16,47 | 500 |
| 7 | TRY-0026  (ACB-Blocks) |  | -7,15 | -2,18 | -48,39 | 500 |
| 8 | TRY-0028  (ACB-Blocks) |  | -7,38 | -4,22 | -49,14 | 640 |
| 9 | TRY-0033  (ACB-Blocks) |  | -7,47 | -4,27 | -50,67 | 400 |
| 10 | TRY-0036  (ACB-Blocks) |  | -6,56 | -4,51 | -50,11 | 500 |
| 11 | TRY-0047  (ACB-Blocks) |  | -5,85 | -6,62 | -43,26 | 500 |
| 12 | TRY-0048  (ACB-Blocks) |  | -6,37 | -4,79 | -52,12 | 500 |
| 13 | TRY-0057  (ACB-Blocks) |  | -7,46 | -4,54 | -49,26 | 500 |
| 14 | TRY-0074  (ACB-Blocks) |  | -7,04 | -4,69 | -55,28 | 260 |

**Synthesis of (5-bromo-1,2-benzisothiazol-3-yl)guanidine hydrochloride.** A solution of guanidine was prepared from 24 ml of absolute ethanol, sodium (0.28 g, 12 mmol), and guanidine hydrochloride (1.24 g, 13 mmol). To 5-bromo-3-chloro-1,2-benzisothiazol (0.50 g, 2.01 mmol) 20 ml of that solution (10 mmol of free guanidine) was added and kept at reflux for 96 h. Ethanol was removed in vacuo, the residue was triturated with dilute NaOH, then brought into 40 ml of boiling methanol, made acidic with conc. HCl, and evaporated to dryness. The remained solid was triturated with hot toluene, then with boiling ethanol, and finally recrystallized from methanol – water (1:1). Yeld 0.48 g (78 %). Spectrum NMR ^1^Н (DMSO-d_6_, 300 MHz): 7.84 d, J=8.0, 1H; 8.21 d, J=8.8, 1H; 8.37 br.s >3H; 8.92 s, 1H; 12.32 s, 1H. Mass-spectrum (direct input, EI): 272+270, 88%, M; 255+253, 100%, M-NH_3_ ; 230+228, 38%, M-NH_2_CN.
